# A solo journey in the shadow of a double-edged pandemic: A qualitative study of women’s experience of being pregnant during the COVID-19 pandemic

**DOI:** 10.1371/journal.pone.0349378

**Published:** 2026-05-15

**Authors:** Ylva-Li Lindahl, Helene Norén, Andrea Hess Engström, Maria Grandahl, Cecilia Åslund

**Affiliations:** 1 Centre for Clinical Research, Västmanland Hospital Västerås, Region Västmanland, Uppsala University, Västerås, Sweden; 2 Department of Women’s and Children’s Health, Uppsala University, Uppsala, Sweden; 3 CHAP, Department of Public Health and Caring Sciences, Uppsala University, Uppsala, Sweden; Deakin University, AUSTRALIA

## Abstract

The COVID-19 pandemic profoundly affected the emotional well-being of pregnant women. In Sweden, no national lockdown was implemented; instead, healthcare restrictions were imposed, most notably limiting partner involvement in perinatal care. This marked a significant shift from standard practices that emphasize partner participation as a key component for maternal support and well-being. This study aimed to explore women’s experiences of being pregnant during the COVID-19 pandemic. A qualitative interview study was conducted in Sweden, including 30 pregnant women. The data were analyzed using content analysis with an inductive approach. Four overarching themes were identified: Living in the Shadow of the Pandemic, Missing Out on the Shared Journey, Unpredictability Creates Worry and Fear, and Adaptation and Growth in a Threatening World. The findings describe pregnant women’s experiences during the COVID-19 pandemic as a period marked by vulnerability, partner exclusion, uncertainty and emotional strain. However, many participants also demonstrated resilience and developed adaptive strategies. A recurring desire emerged for more inclusive, consistent healthcare practices and clearer communication tailored to the needs of expectant families. The findings highlight the importance of patient-centered, accessible perinatal healthcare and contribute to the existing research by emphasizing the role of support systems and national guidelines in safeguarding maternal and family well-being during future pandemics.

## Introduction

The COVID-19 pandemic had a major impact on the entire society and healthcare systems globally [1]. Governments around the world introduced various measures to prevent the spread of the virus, including restrictions and lockdowns [2]. While these measures were instrumental in reducing transmission rates, they also had unintended negative effects on perinatal mental health. Pregnant women experienced increased stress and anxiety due to restricted access to healthcare and the exclusion of partners during critical moments of the perinatal period [3].

Unlike many countries, Sweden adopted a distinct approach by prioritizing recommendations and voluntary compliance over mandatory lockdowns [4]. However, Swedish healthcare imposed certain restrictions to mitigate COVID-19 risks [5], although these varied across the country due to regional autonomy and differences in healthcare resources [6]. A key element regarding restrictions in perinatal healthcare in Sweden was the limited involvement of the partner [7], marking a significant deviation from standard practices in Sweden, where partner support during pregnancy is considered vital to maternal well-being [8]. Sweden’s strong commitment to gender equality in parenting is reflected in its generous parental leave policies and longstanding societal expectations of shared parenting and high partner involvement. Thereby, restrictions in perinatal healthcare, particularly limited partner involvement during antenatal- and postnatal healthcare and childbirth, represented a significant shift. Such changes may have important implications for expectant parents, especially in a context where partner support is considered central to maternal well-being [7,8].

The pandemic heightened pregnant women’s concerns about potential risks to both maternal and fetal health, as well as their access to healthcare services [9–12]. Studies indicate that the emotional well-being of pregnant women was adversely affected by the pandemic, with increased anxiety due to uncertainties about COVID-19’s impact on pregnancy [13–15]. Data from multiple countries, including China, Japan, Turkey, and the United States, revealed significant increases in anxiety and depression among pregnant women, with some reports indicating rises of 25–50% in depressive and anxiety symptoms [9,14,16–21]. In addition to its impact on pregnant and postpartum women’s mental health, the pandemic also led to an increased use of coping strategies [16,22–24]. These elevated mental health issues were exacerbated among individuals already at higher risk for mental health difficulties prior to the pandemic [5]. Additional factors influencing perinatal mental health included ability for resilience, educational level, gestational trimester, and ethnicity [25]. Depression and anxiety during pregnancy have profound implications for maternal and fetal health, as they are associated with increased risks for adverse psycho-emotional developmental outcomes in children [26,27]. Furthermore, pandemic-related stress and anxiety did not universally impair maternal-fetal bonding; rather, symptoms of depression appeared to be a more significant barrier to bonding [28]. Women reported feelings of loss over missed pregnancy experiences and had concerns related to birthing uncertainties [22].

Pregnancy is a unique transitional period marked by psychological adjustments and varying emotional experiences, with each woman encountering motherhood in distinct ways [29]. Research underscores the importance of the partner’s involvement during this time, as partner support is linked to reduced maternal stress and improved mental health [8,30].

When this study was initiated, the COVID-19 pandemic represented a novel global health crisis, and research on its impact on pregnancy and the perinatal period was still emerging. Early studies from Sweden indicated that pregnant women and their partners experienced increased uncertainty, altered support structures, and disruptions in maternity care [31,32]. However, in-depth qualitative evidence remains limited, particularly regarding how women experienced pregnancy within specific healthcare contexts during the pandemic. A deeper understanding of these lived experiences is needed to capture the complexity and variability of pregnancy during a rapidly evolving public health situation. Therefore, the aim of the present study was to explore women’s experiences of being pregnant during the COVID-19 pandemic.

## Materials and methods

### Study design

In this interview study, we used qualitative content analysis with an inductive approach [33–35]. The study follows the Standards for Reporting Qualitative Research and is reported according to the COREQ Checklist [36] (S1 File).

### Setting

In Sweden, the population is approximately 10.6 million (2025), and there are 21 regions [37]. The healthcare system is primarily funded by regional governments. There is regional autonomy, and the healthcare services can be designed according to regional conditions [6]. Residents of Sweden have general access to publicly funded hospital care and primary healthcare, as well as maternal healthcare and child healthcare. Healthcare during the perinatal period is provided free of charge for all women and newborn infants. The study was conducted in Västmanland Region, a medium-sized region in central Sweden with approximately 280 000 inhabitants. In 2024, there were 98 451 births in Sweden, of which 2 568 occurred in the Västmanland Region [37]. The region includes 25 maternity clinics and one hospital with a maternity ward [38].

### Study population

Individuals aged 18 years or older who were pregnant and participating in an ongoing quantitative questionnaire study were eligible for inclusion. The exclusion criterion was an inability to communicate in Swedish. All 479 individuals who had completed a questionnaire with questions about the pandemic were invited via mail. A total of 145 indicated their willingness to be contacted and provided their phone numbers. Participants were then approached consecutively by phone. Those who answered received a formal invitation to participate and information about the study. Those who opted to participate were subsequently scheduled for an interview and they received written information and a consent form by post, which they signed prior to the interview. In total, 30 pregnant women were included in the study. No one declined participation or dropped out during or after the interviews. Their ages ranged from 23 to 39 years, with a mean age of 31.2, and a median age of 31.5 years. Sixteen women had given birth previously, while fourteen were first-time mothers. No reimbursement was offered. Regarding demographic characteristics, participants in the interview study were broadly similar in age but had slightly higher educational attainment compared to non-participants.

We acknowledge that not all pregnant women who give birth identify with the term “woman”. However, for the purpose of this study, we have used the term “woman” to describe our participants, as this terminology is consistent with and aligns with the existing literature.

### Data collection

The interviews were conducted digitally (Microsoft Teams™) between September 2020 and July 2021 by the authors (Y-LL, PhD student, coordinating midwife/healthcare developer, and HN, PhD student, perinatal psychologist). During the interviews, no individuals other than the participant and the interviewers were present. A few field notes were made during the interviews; however, they were not used in the analysis. The interviews lasted approximately 40 minutes (range 22–62 minutes) and were recorded by using an external dictaphone. No interviews were repeated. Interviews were conducted until no additional dimensions or variations emerged. The sample size was continuously assessed based on the study’s information power, ensuring sufficient data to address the research question [39].

### Interview guide

The interview guide (S2 File) was developed by the authors and subsequently reviewed by experts in psychology. No pilot testing was conducted prior to the interviews. The interview questions focused on experiences of being pregnant during the pandemic, perinatal healthcare, the relationship with one’s partner, contact and support from relatives and friends, information from authorities and healthcare providers, and media reporting related to the pandemic. Follow-up questions were posed when necessary, allowing participants the opportunity to elaborate further on their responses.

### Data analysis

The recorded interviews were transcribed verbatim. The transcripts were not returned to the participants for comment or correction, and member checking of the findings was not conducted. The whole context of the text was considered throughout the analytical process. The transcripts were read repeatedly to gain an overall picture of the data and obtain a comprehensive understanding. Initially in the analysis process, meaning units were identified, condensed and subsequently labelled with codes that stayed close to the manifest content of the text, according to the aim of the study. In the next step, the codes were systematically compared within and across interviews regarding similarities and differences and then sorted into subcategories reflecting shared characteristics [33]. Then the sub-categories were compared with similar manifest content. Categories were developed to describe the manifest content, with a low level of abstraction and interpretation. The themes represented a higher level of abstraction, capturing the underlying meaning and forming a ‘red thread’ across categories. They brought meaning to the phenomenon under study and its various manifestations and expressed the latent content of the data as a whole [34,35]. The analytic process was iterative, involving continuous comparison between data, codes, and developed categories. Saturation was operationalized as both code saturation and meaning saturation. Code saturation was reached when no new codes emerged in the analysis, while meaning was achieved when the categories and themes were sufficiently rich and captured the variation and depth of the data. The category describes the content on the manifest level, with a progressively varying degree of abstraction. Interpretation entails moving from descriptions of the manifest content to interpretations of the latent content. The analysis continued until no new categories or themes emerged and the data provided a comprehensive understanding of the phenomenon.

In order to avoid researcher bias, three authors (Y-LL, HN, CÅ) read the transcripts and performed the analysis. The analytical process was dynamic, involving multiple iterations. The analysis was validated by MG and AHE to enhance trustworthiness [40]. After review and discussion within the research team, the categorical structure was assessed to be stable, and data analysis was consequently concluded. The computer program Excel™ (Microsoft Inc., WA, USA) was used to manage the data.

### Reflexivity

The authors (Y-LL, HN) who conducted the interviews were employed in the regional central maternal health care team and have clinical experience in maternal and mental health care. Their professional backgrounds provided contextual understanding of pregnancy and psychological well-being, which facilitated rapport and in-depth discussions with participants. However, their clinical experience may also have influenced the interview situation, including the framing of follow-up questions, the interpretation of participants’ narratives, and sensitivity to certain topics. The interviewers therefore engaged in continuous reflection on their pre-understandings and professional perspectives, particularly regarding maternity care during the pandemic [34,35]. Reflexivity was operationalized through repeated reflection on the preconceptions and discussions within the research team. This collaborative and reflective approach aimed to enhance transparency and credibility in both data collection and analysis.

### Ethical considerations

The study was approved by the Swedish Ethical Review Authority (Number 2020−03479; Number 2018−332) and conducted in accordance with the Declaration of Helsinki. Participants received verbal and written information about the study and were informed that participation was voluntary, and that they could withdraw at any time, for any or no given reason, without incurring any negative consequences for themselves. They were also informed that only the researchers would have access to the data and that all data would be presented at group level. Contact details for the researchers were provided in case of further questions. Confidentiality was ensured, and the participants’ personal data was pseudonymized, with names replaced by codes, and the participants were referred to as *Informant 1–30* after inclusion.

## Results

The findings in this study describe women’s experiences of being pregnant during the COVID-19 pandemic. During the analysis process, four themes were identified that summarize the results: Living in the Shadow of the Pandemic, Missing Out on the Shared Journey, Unpredictability Creates Worry and Fear, and Adaptation and Growth in a Threatening World. Based on these four themes a synthesis was developed; A solo journey in the shadow of a double-edged pandemic, to highlight their integration into a cohesive understanding. The term “double-edged pandemic” refers to the dual nature of participants’ experiences during COVID-19. While participants described the pandemic as a challenging period, they also reported positive aspects, including personal growth, increased resilience, and strengthened social connections.

**Fig 1:**
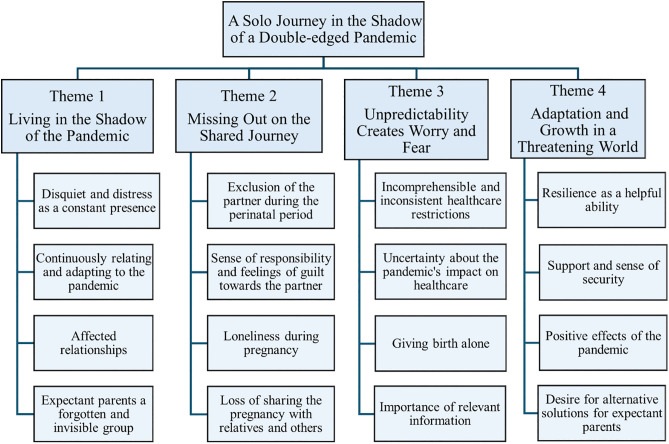
An overview of the themes and categories. The four themes that summarize the results are presented with the underlying categories. Based on these themes, the synthesis A solo journey in the shadow of a double-edged pandemic is presented at the top, illustrating their integration into a cohesive understanding.

The results are presented in text with the themes as headings and the categories as subheadings. The content of the subcategories is summarized in the paragraph by respective categories in the results section. Illustrative quotes are presented in connection with the themes and categories to exemplify the informants’ experiences. An overview of the themes and categories is presented in Fig 1.

### Theme 1: Living in the Shadow of the Pandemic

The informants described the pandemic as having affected them in several ways, similar to the broader population. They emphasized the particular challenge of the pandemic occurring during pregnancy, a crucial life stage, and highlighted the various ways in which perinatal healthcare was impacted. The pandemic remained a constant backdrop to their experience, with a primary focus on avoiding infection for both the pregnant woman and her partner. This theme is structured around the following categories: Disquiet and distress as a constant presence; Continuously relating and adapting to the pandemic; Affected relationships; and Expectant parents a forgotten and invisible group during the pandemic.

#### Disquiet and distress as a constant presence.

The informants described how the pandemic affected the entire world, with many living in fear of becoming seriously ill. At the onset of the pandemic, there was significant uncertainty and ambiguity regarding how COVID-19 might affect pregnant women and their fetuses. This uncertainty contributed to a persistent sense of unease, with a constant need to remain vigilant in avoiding infection, which in turn led to feelings of disquiet and distress. They described the additional emotional burden of monitoring their partner and any designated back-up individuals for signs of infection, given the potential impact on the partner’s ability to participate during labor. This vigilance extended to monitoring relatives for symptoms to ensure they could provide childcare for older siblings during labor, necessitating constant re-evaluation of family members’ health status. The potential for the partner to contract the virus at work or for children to be exposed at preschool or school led to concerns and heightened vigilance, with continuous monitoring whenever any family member exhibited symptoms.

*“It’s an underlying stress, that you don’t really know how much it’s spreading in the general population, do you dare go out, are any activities available?... That underlying stress is more about always looking out for symptoms:‘Yes, but do they have runny noses?’ And myself…’is it the pregnancy that’s giving me a bit of a headache? Or am I starting to come down with a cold?’”*

*# Informant 5*

The participants expressed concerns that the timing of their pregnancies during the pandemic may have been less than optimal. They also reflected on whether it had been wise to conceive during such uncertain times and, upon becoming pregnant, whether they should proceed with the pregnancy.

*“We’ve been very worried during this whole process, and yes, this sounds terrible, but we were considering whether we should risk going through with the pregnancy or not because of the situation.”*

*# Informant 13*

#### Continuously relating and adapting to the pandemic.

The informants described their life situation during the pandemic as marked by a profound sense of loss, particularly the loss of freedom in daily life. They expressed a longing for the ability to engage in ordinary activities, such as shopping or meeting friends at a café, without the accompanying worry and the restrictions imposed by the pandemic. Additionally, the limitations on opportunities to undertake practical preparations for the arrival of their baby were highlighted as a significant source of frustration and concern.

*“Perhaps I would have liked to go around the shops looking at baby clothes or go buy some things I would need. It was obviously more complicated...The practical preparations were more difficult.”*

*# Informant 24*

These reflections underscore the pervasive impact of the pandemic on both personal and social dimensions of life. The informants emphasized concerns about the risk of infection at the maternity clinic or hospital. Additionally, they described implementing preventive measures within the household to minimize the risk of viral transmission, reflecting precautionary behaviors necessitated by the pandemic.

*“In the labor ward. Because in that hospital environment, with all the staff there, there are or have been people infected with corona… in that hospital environment with potentially more infections, so that’s also a risk.”*

*# Informant 3*

##### Affected relationships.

The informants reported that the pandemic significantly affected their relationships with partners, relatives, and friends. Isolation and the shift to working from home introduced particular strains, especially for couples who shared a home workspace. This new dynamic often resulted in increased irritability and challenges in maintaining harmony. For individuals working from home alone, the isolation was described as equally challenging, amplifying feelings of loneliness.

*“And then maybe we’ve been having more rows, or... been... maybe I’ve been more difficult myself... And then, I suppose I don’t think that it has in the long-term... there were so many little bullshit things that irritated me, like.”*

*# Informant 30*

They also expressed a deep longing for physical closeness to their relatives, such as being able to hug their parents, which was notably absent during the pandemic. Relationships with friends were also impacted, despite efforts to stay connected through social media and phone calls. Additionally, they described a sense of loss in their relationships with friends’ children, noting that they missed witnessing and engaging with their childhood experiences. These findings highlight the complex and multifaceted effects of the pandemic on social and emotional connections.

*“Especially the social side of things, that’s the first bit… that I can’t see my loved ones the same way. I can’t hug my mum or my family the same way*. *And that I can’t be with my friends.”*

*# Informant 14*

#### Expectant parents a forgotten and invisible group.

The informants highlighted the perinatal period as both a vulnerable and crucial time, significant for the entire lifespan. It was described as a special event that occurs once or perhaps a couple of times in life. They also emphasized the postpartum period as a time of major adjustment and additional vulnerability for women, noting the need for support from their partners. They stressed that the experiences of pregnant women and expectant parents during the pandemic were largely overlooked.

*“I don’t think there is very much [information]. They might not know so much either, I don’t know, but there hasn’t been very much focus either, that’s the way it feels.”*

*# Informant 25*

They described how authorities focused on the need to protect the elderly and frail from infection, and even patient groups with chronic diseases were considered particularly vulnerable to COVID-19. However, pregnant women were largely overlooked, and it remained unclear whether they were classified as a high-risk group. Measures were introduced to protect pregnant women and their fetuses, including the suspension of work for those with occupational exposure to COVID-19 in the workplace. These factors complicated the pregnancy experience, and the informants felt that these issues were neither addressed nor represented in the media. They described a sense of belonging to a forgotten and invisible group during the pandemic.

*“It’s been a bit like, ‘Well, what are the restrictions now?’. You have to read up about it yourself, like, because I can’t just assume that I’ll be told what applies to me as a pregnant woman, as much as being told that these are the restrictions for old people. The elderly are a rather permanent group, or…well it’s not temporary, you belong to that group.”*

*# Informant 23*

### Theme 2: Missing Out on the Shared Journey

The fact that partners’ participation in perinatal healthcare was limited during the pandemic emerged as a consistent theme throughout the interviews. This theme is structured around the following categories: Exclusion of the partner during the perinatal period; Sense of responsibility and feelings of guilt towards the partner; Loneliness during pregnancy; and Loss of sharing the pregnancy with relatives and others.

#### Exclusion of the partner during the perinatal period.

The informants consistently described the exclusion of the partner during the perinatal period as a significant and distressing consequence of healthcare restrictions implemented during the pandemic. Partners were often prohibited from attending key moments, such as visits to the maternity clinic, ultrasound examinations, and postpartum care at hospitals. This exclusion was consistently characterized in the interviews as a source of sadness, sorrow, and frustration, with informants lamenting the loss of these shared experiences and the inability of the partner to fully participate in this pivotal period. The presence and support of the partner during the perinatal period were emphasized as critical for the well-being of the pregnant woman. Informants highlighted the importance of the partner’s presence in fostering a sense of security and reinforcing the couple’s partnership, underscoring their interdependence during this transformative time. Furthermore, the exclusion was seen as a missed opportunity for the partner to prepare for and engage with the birth of their child, a process viewed as essential for establishing a strong bond and relationship with the baby.

Concerns were also expressed regarding the long-term effects of this exclusion on both the partner and the child, with informants reflecting on the potential impact on familial relationships and the partner’s ability to form a deep connection with the baby. These insights illustrate the profound emotional and relational consequences of partner exclusion during the perinatal period. The informants emphasized the importance of the partner’s presence during ultrasound examinations, highlighting various perspectives on its significance. Ultrasound was described as a crucial moment for the partner to experience the baby’s first signs of life, promoting emotional connection and shared anticipation of parenthood.

*“It felt a bit strange because it was like the first actual sign of life, you get to see the baby. And we had waited such a long time for this, so it was rather mixed feelings to experience it on my own.”*

*# Informant 16*

Ultrasound serves as a form of fetal diagnosis, and the informants expressed concerns about the possibility of receiving difficult or distressing news alone in the absence of their partner. This absence was perceived as emotionally challenging and detrimental to the shared support and unity of the couple during such critical moments.

*“Let’s say that something turns out to be wrong with the baby, something isn’t right, or that there are twins for that matter, or something that could be... well, that you can be a bit shocked by that information, and to have to cope with that on your own. I haven’t just assumed that the baby will be healthy, like, I’ve been quite aware that there could be things that turn up during the scan.”*

*# Informant 21*

The crucial importance of the partner’s presence during childbirth was underscored, particularly in the final months of pregnancy. Concerns centered around the possibility that partners might be prohibited from attending the birth if they exhibited symptoms associated with COVID-19. The informants highlighted the profound significance of the couple sharing the immediate postpartum period with their newborn, a time they viewed as essential for bonding and mutual support. The inability to share the perinatal experience as a team was described as a deeply felt loss that affected their emotional well-being. The informants also reflected on how this experience might differ for first-time parents compared to those who already had children, suggesting that previous parental experience may influence the coping strategies and perspectives of the couple.

#### Sense of responsibility and feelings of guilt towards the partner.

The informants described a profound sense of responsibility arising from the exclusion of partners from perinatal healthcare during the pandemic. This responsibility included maintaining communication with midwives at the maternity clinic and ensuring that their partners were kept informed about essential aspects of pregnancy, childbirth, and parenthood. One informant likened this role to “being the spider in the web in relation to healthcare,” underscoring the central role they played in bridging the gap between healthcare providers and their partners.

“*I feel that I’ve had to do more explaining to him than if there hadn’t been a pandemic. He hasn’t been offered anything by healthcare services... there haven’t been any groups or anything. So, I’ve been the one who had to explain to him. I’ve been, like, the main contact person when it came to healthcare, and then I have to pass it on to the third party, that is, to him.”*

*# Informant 2*

The informants also expressed significant concern about their partners’ experiences and feelings of exclusion during this period. This exclusion was perceived as an inequity that marginalized the partner’s role as an expecting parent. Many informants articulated feelings of guilt for being the sole recipient of healthcare services and support, while their partners were deprived of these experiences. These reflections highlight the emotional strain and ethical dilemmas faced by expectant mothers as they navigated the dual responsibilities of accessing care and compensating for their partners’ exclusion during the perinatal period.

*“Feelings of guilt and responsibility and quite a large internal debate going on* *inside me, whether I should stay at the postnatal ward or not, afterwards... If I choose to go home early, we can be together, my partner will also get to [connect with the baby] right away, it’ll be more equal... In a way it felt egoistic to stay [in the postnatal ward].”*

*# Informant 3*

#### Loneliness during pregnancy.

The informants reported experiencing loneliness in relation to healthcare during pregnancy. They described a pervasive sense of isolation during the pregnancy overall; however, many also noted that they eventually became accustomed to being alone and had to engage in more solitary activities. To minimize the risk of infection prior to childbirth, they chose to self-isolate, which further contributed to their feelings of loneliness, particularly during the transition to remote work.

*"I guess it’s about that I’ve been very alone in it all, that’s what I would say. I think there’s generally a lot to deal with, both this, how you feel and when you’ve been working from home and the pregnancy, and there’s a lot of loneliness for a lot of people, for me too, when I’ve been working alone at home.”*

*# Informant 30*

#### Loss of sharing the pregnancy with relatives and others.

A particular concern was the potential impact on the baby’s social relationships, with some informants expressing worry that their child might become forgotten within the broader network of family and friends. The informants expressed a profound sense of loss regarding the inability to share the experience of pregnancy with relatives and friends due to pandemic restrictions. They highlighted the limited opportunities for their social circle to engage with the pregnancy and to form meaningful connections with the unborn child.

*“We started to pluck up the courage to spend more time with others at the beginning of autumn, but […] I know my siblings said that they essentially haven’t seen (laughs) this pregnancy at all.”*

*# Informant 19*

The informants reported a longing to participate in parent groups with other expectant parents at the maternity clinic. These activities were cancelled during the pandemic, and expectant parents were offered alternative resources, such as websites or midwife-facilitated online groups and this virtual alternative did not adequately substitute for in-person interactions.

*“Your first pregnancy that you really… or that I really would have wanted to share with more people, you know, share while being physically together [……] It’s such a shame, I mean both the parental education (sighs) and childbirth preparation. We’ve moved to our town relatively recently, and it would have been a way to… we had been looking forward to meeting other parents in the same situation, in order to create a social network also.”*

*# Informant 3*

### Theme 3: Unpredictability Creates Worry and Fear

The informants described the pandemic as a new and unfamiliar situation, and the scenario was unpredictable, which led to uncertainty. This theme was structured around the following categories: Incomprehensible and inconsistent healthcare restrictions; Uncertainty about the pandemic’s impact on healthcare; Giving birth alone; and Importance of relevant information.

#### Incomprehensible and inconsistent healthcare restrictions.

The restrictions in perinatal healthcare were described as incomprehensible when compared with those applied to the rest of society. The recommendations from authorities and the adaptation of information changed rapidly due to the evolving nature of the pandemic. This led to feelings of insecurity and questions such as, “Am I doing the right or wrong thing?” The informants expressed that the healthcare system should have handled the interpretation and implementation of restrictions differently to enable partner participation in healthcare during the perinatal period. The restriction on partner involvement was perceived as outdated and inequitable, failing to acknowledge that the partner is also becoming a parent.

*“They’ve gone back to the sixties, like, ‘The partner isn’t worth anything, he isn’t going to be a parent, you’re the parent, full stop.’ So, it feels very outdated somehow, how they’ve handled the whole corona situation. Also, somehow, that there should be more understanding that he’s also going to be a parent.”*

*# Informant 17*

They noted that restrictions in perinatal healthcare differed across regions in Sweden, which led to frustration, uncertainty, and concerns about giving birth in another region. They also described the rationale behind the decisions as vague and unclear, expressing a need to understand the background and the authorities responsible for making these decisions.

*“And I haven’t gotten an answer to why... who actually made the decision, that your partner can’t go with you to the postnatal ward, even though he can go with you to the labor ward. Has this been discussed with specialists in this area or is it a regional Västmanland politician that made the decision and... without consulting specialists in the area?”*

*# Informant 8*

It would have been more understandable if all regions in Sweden had adhered to national guidelines, thereby promoting greater equality and avoiding inconsistent restrictions. Furthermore, the informants noted that society interpreted the recommendations from authorities differently, which sometimes led to conflicts in workplaces or within families.

#### Uncertainty about the pandemic’s impact on healthcare.

Many of the pregnant women reported that antenatal care deviated from routine practices, appearing more perfunctory and procedural in nature. The informants expressed uncertainty about whether the healthcare system would be sufficient to meet their needs and capable of prioritizing women in labor in relation to patients who were seriously ill with COVID-19.

*“Maybe you’re not such a priority when you’re pregnant... the hospitals are full of people that are seriously ill with COVID and are dying. That’s when you feel like, ‘Yes, but me, (laughs) I’m just here to have a baby’.”*

*# Informant 23*

They also expressed concerns about the potential shortage of healthcare professionals if the healthcare staff were to fall ill as well. Further apprehensions were linked to the availability of personal protective equipment for healthcare providers, with participants worrying that inadequate protection might increase the risk of infection for both staff and patients. Many participants feared being unable to establish a meaningful connection with healthcare professionals due to the perceived depersonalization of care and the presence of physical barriers.

*“When they start to talk about masks for visitors, I think it would be incredibly difficult to give birth wearing a mask. Because you get, like, a feeling of being suffocated, and then you’re meant to lie there for maybe 24 hours and try to give birth to a baby and be feeling that you’re being smothered the whole time...[….]..”*

*# Informant 24*

Additional concerns centered on the potential risks to the newborn if the mother were to contract COVID-19 during labor, as well as the uncertainty about how the healthcare system would respond to such situations. The sense of guilt and fear of being a nuisance to the professionals, who had extreme and strained working conditions, also emerged as a recurring theme in the interviews.

#### Giving birth alone.

The informants consistently described the emotional turmoil caused by the uncertainty regarding their partners’ presence during labor, and the prospect of giving birth under such uncertain circumstances was highly distressing. Hospital policies, often subject to sudden changes, dictated whether partners could attend, leaving many individuals feeling unprepared, vulnerable, and unsupported. Even minor symptoms, such as a cough or low-grade fever in the partner, could lead to their exclusion, intensifying the anxiety of an already challenging experience.

*“Not terrified when it comes to me having serious complications and so on, but that I’ll infect my partner. I hope my lungs can handle a COVID-19 illness. But it’s more that I would... infect my partner who wouldn’t be allowed to be there when I give birth… Because that’s something I don’t want to experience on my own. I don’t want to be in the labor ward alone... or with some other family member than my partner.*

*# Informant 14*

This uncertainty was reported as having significant psychological consequences, heightening feelings of isolation and vulnerability during a time when emotional support is crucial. Some informants reported a pre-existing fear of childbirth prior to the pandemic, while for others, the ambiguity surrounding partner attendance gave rise to entirely new fears—even among those who had previously approached childbirth with confidence. These feelings of unease were not limited to first-time parents but were also experienced by individuals who had previously given birth, underscoring the unique stressors associated with the pandemic context. The uncertainty of giving birth alone in that situation was consistent in the interviews and was described as deeply distressing and terrifying.

*“I think the word ‘worry’ is too small to use, because I feel… yes, it’s a huge worry, a huge… yes, I would almost… well, maybe not terror either, but something in between.”*

*# Informant 27*

#### Importance of relevant information.

The informants attempted to search for information but found it difficult to locate resources specifically tailored and adequate for pregnant women. They relied on information from friends who were also pregnant or on rumors about healthcare restrictions on social media.

*“I think it’s more that, like, all the stuff that’s been written has affected me, as many other people picked up things and said things to me, like, ‘Have you heard that it’s like this now? And did you read that?’”*

*# Informant 21*

There was also a perception that the media engaged in scaremongering and were seen as unprofessional and lacking credibility. However, they felt that, at times, there was an overwhelming amount of information, which they described as “chaotic noise”. It was difficult to sift through the information, with numerous possible interpretations.

*“Many media write headlines that are meant to scare you. And I’ve tried both to read it, but to still disregard some things, just that the headlines are meant to be scary, and I’ve tried to watch more Swedish public service TV and read DN [major daily newspaper]… where it’s a bit more neutral”*

*# Informant 17*

Despite this, the women expressed that receiving some information was still preferable to having no information at all. At the same time, the informants were satisfied with the general information about the pandemic provided by Swedish authorities. There was a clear need for uniform, consolidated information from healthcare authorities, specifically tailored to pregnant women and their partners regarding current restrictions.

### Theme 4: Adaption and Growth in a Threatening World

Despite the numerous negative consequences of the pandemic for expectant parents, the informants described, from several perspectives, how they dealt with the aggravating circumstances. This theme was structured around the following categories: Resilience as a helpful ability; Positive effects of the pandemic; Support and sense of security; and Desire for alternative solutions for expectant and new parents.

#### Resilience as a helpful ability.

The informants reflected on the COVID-19 pandemic as an unprecedented and unique situation, highlighting the initial absence of research and knowledge regarding its impact on pregnancy. They demonstrated an acceptance of this uncertainty, recognizing that the lack of information was an inevitable consequence of the novel nature of the virus. Despite the many difficulties brought about by the pandemic, the informants described their ability to navigate these challenges as being influenced by their personality and previous experiences with adversity. Those who had received useful guidance in relation to previous mental ill-health expressed that it was helpful in this situation and found these coping strategies beneficial in the context of the pandemic. Moreover, the informants expressed an understanding of the need for restrictive measures and conveyed their trust that healthcare professionals were doing their utmost to provide optimal care under the circumstances. This acknowledgment of healthcare workers’ efforts, coupled with their own resilience, enabled the informants to face the challenges of the pandemic with composure and adaptability.

*“…I went there [the scan] and he didn’t, and maybe he would have wanted to once, to come with me in order to listen to the baby’s heartbeat. But then he could also feel it moving, and that almost meant more actually.”*

*# Informant 15*

They also expressed a high level of acceptance of the pandemic’s challenges and empathy for healthcare professionals, acknowledging their workload and constraints. Informants reported employing various strategies to shield themselves from the negative impact of media coverage, coupled with an understanding that the situation was beyond their control. Informants described how they were navigating pregnancy during the pandemic, noting that their attitudes and approaches were effective in managing the situation. Additionally, the anticipation of the baby was highlighted as a protective factor, providing emotional relief and positivity amid challenging circumstances and serving as an important coping mechanism.

*“My own coping with pain, how I’ll manage it, and so on. But then I trust healthcare and healthcare staff to an extreme extent, that they’ll help me and so on, and that also makes my fear... the fear has really gone down a whole lot, and as I said before, looking forward more to the baby also made me less scared.”*

*# Informant 17*

#### Support and sense of security.

The informants emphasized the pivotal role of midwives in antenatal healthcare, particularly in providing support that significantly enhanced the sense of security among expectant parents. Midwives were recognized for their ability to foster trust and reassurance through their professional expertise and empathetic approach. In some cases, midwives implemented individualized solutions to facilitate the involvement of partners in antenatal care, even amidst restrictive circumstances. These tailored measures were described as highly valuable and contributed to a more inclusive experience. The sense of trust in the healthcare services provided during childbirth and the postpartum period was a significant source of reassurance for expectant parents.

*“I choose to believe that the necessary staff and resources will be available there at the labor ward, regardless of the corona situation, despite the exhaustion that I’ve heard the staff has had after the spring and summer. But I also think that the staff are super-competent, so that everything will be ok.”*

*# Informant 3*

Experienced mothers expressed a sense of security about the prospect of being alone during childbirth, citing previous experience and familiarity with the process as key sources of confidence.

*“I’ve gone through a delivery before, so I know how important a partner’s support is, but at the same time I know that there are very competent staff in the labor ward.”*

*# Informant 16*

However, they also expressed empathy for first-time parents who faced the added emotional and logistical challenges of navigating childbirth under similar conditions. Informants who participated in online parental support groups and engaged in virtual sessions with perinatal psychologists reported positive experiences and expressed appreciation for these digital services, which were particularly valued during the pandemic’s restrictive circumstances. Additionally, virtual meetings with friends who also had children were described as helpful for sharing experiences.

#### Positive effects of the pandemic.

Despite the challenges imposed by the COVID-19 pandemic, the informants reported that they were able to identify several positive aspects arising from the circumstances. One notable benefit was the opportunity to rest while working or studying from home, a flexibility that proved especially advantageous for individuals experiencing pregnancy-related physical discomforts. This arrangement allowed some to remain professionally active in their pregnancies longer than might have otherwise been possible, providing a significant advantage in terms of both career continuity and personal well-being.

*“In that case, I probably would have had to go on leave, and so I can see that it was more positive to work from home. I want to work, you see.”*

*# Informant 30*

The restructured daily routines during the pandemic also facilitated shared responsibilities within couples, particularly in managing childcare and preschool drop-offs, as both partners were able to coordinate and support each other more effectively from home. Additionally, for those who commuted, it provided relief as the commuting time was reduced. The opportunity to spend more time together with the family or as a couple was also highly appreciated by the informants. The increased time at home was also positively associated with family and relationship dynamics. The relationship was described as improving and becoming stronger, developing into a sense of “we against the world”. This period served as an affirmation of the positive atmosphere in the home and strengthened relationships, which many informants described as being significantly enhanced during this time. An additional positive outcome of remote work was the reduced exposure to unsolicited advice on pregnancy and parenting from others, which some informants found to be intrusive and unhelpful. Working from home created a buffer that allowed them to navigate pregnancy and early parenting with fewer external pressures, contributing to a more autonomous and peaceful experience.

*“Then I can divide up my day in another way, so I have time to rest, and I have time to work, I have time to get things done, more then I might have had if there hadn’t been corona. I would have been at work much more. I think I might have been more tired in that case.”*

*# Informant 5*

#### Desire for alternative solutions for expectant parents.

The informants expressed a strong desire for perinatal healthcare to handle restrictions, and they advocated for personalized and flexible measures to facilitate greater involvement of partners during the perinatal period. Several alternative solutions were proposed to mitigate the exclusion of partners, including the implementation of special adaptations that consider the unique needs and circumstances of expectant and new parents. For example, specific suggestions involved designated time slots for partner presence, digital engagement options for situations where physical presence might be constrained, and enhanced communication to ensure partners remain informed and involved.

*“Why don’t they just do everything they can so that the dad can be part of it? Especially when it’s your first baby. I think it’s very important when it comes to the second and third baby also, but… most of all the first.”*

*# Informant 10*

## Discussion

This qualitative study explored women’s experiences of being pregnant during the COVID-19 pandemic, highlighting key emotional and psychological themes. The results showed multifaceted aspects and several dimensions of being pregnant during the pandemic. Participants expressed feelings of loneliness and the sense of missing out on the shared journey due to the exclusion of their partners in perinatal healthcare. The pervasive unpredictability of the situation was a source of worry and fear, contributing to heightened disquiet and distress. On the other hand, they also reported a capacity for adaptation and personal growth despite the ongoing threats posed by the pandemic. This ability for resilience appeared to be a beneficial attribute. Previous studies underscore resilience as the dynamic interplay between vulnerability and adaptation in navigating an uncertain world. Our findings align with prior research in this area [14,31,32,41,42].

The restriction on partners’ presence in perinatal healthcare had an impact on the expectant parents’ experiences, both in the short and long term. A crucial event during this period was the partner missing the opportunity to share the first days in the hospital with the newborn. This was highlighted from several perspectives as a concern regarding whether the partner could develop a relationship with the newborn. These perspectives and their consequences for expectant parents did not emerge from authorities or the media, leaving them as an invisible group in the shadows. Decision makers did not recognize that the perinatal period is a special time in life, characterized by increased psychological adjustment and vulnerability. Viaux et al. highlighted that reducing psychological vulnerability when adapting essential care practices is valuable for supporting mother-infant bonding and family development during this critical period [3]. The partner was regarded as an extended relative rather than an equal expectant parent. Studies also report the consequences of the exclusion of the partner and suggest solutions for future pandemics [31,32].

In general, expectant parents need greater involvement of the partner, and they employ different strategies to overcome the barriers that they encountered while navigating this unique experience of becoming parents. The best support for the partner often comes from the expectant mother, and healthcare services need to include the partner to a greater extent, focusing on the well-being of the whole family [30]. Lack of support may act as a vulnerability factor for the pregnant woman [43]. Emotional support during pregnancy is associated with increased perceived and physiological resilience, which subsequently predicts fewer issues in offspring [44]. Pregnancy can be a time of deep psychological development and growth, especially when the person receives support in processing the changes and challenges that arise [29]. Swedish perinatal healthcare has developed over several decades and, under ordinary circumstances, actively includes the partner during the perinatal period. In our study, the pregnant women emphasized the importance of the partners’ participation during the process.

Perinatal healthcare restrictions at maternity clinics and hospitals were described as incomprehensible, particularly when contrasted with the broader, less restrictive measures in place across other sectors of society. Informants expressed frustration over the perceived lack of transparency surrounding these decisions, as they struggled to understand the rationale behind the restrictions. The policies appeared vague and unclear, leading many to interpret that there was insufficient justification or evidence underpinning these measures. This underscores the importance of providing adequate information, with the understanding that some information is better than none. It is important that healthcare providers understand pregnant women’s unique experiences in order to support them effectively during upheavals like a global pandemic. By acknowledging the loss of the typical pregnancy experience, offering creative social support, and fostering optimism, healthcare providers can help promote positive outcomes [22]. During the pandemic, the midwives showed understanding for the expectant parents, but they were not involved in the decisions regarding the restrictions. The pregnant women described that the professionals did their best based on the prevailing circumstances.

The pervasive unpredictability of the COVID-19 pandemic was a source of worry and fear, contributing to heightened disquiet and distress among the pregnant women. However, they also demonstrated the ability to adapt and achieve personal growth despite the ongoing threats posed by the pandemic. This resilience underscores the dynamic interplay between vulnerability and adaptation in navigating an uncertain world, and it is in line with previous studies [45]. An indication of increased media consumption is that it is related to increased anxiety symptoms. In order to cope, it is useful to adopt self-care strategies to a greater extent, such as adjusting media consumption to reduce stress [24]. In our study, the participants described that they actively avoided media to reduce anxiety. Active coping strategies and positive experiences can be particularly beneficial for women experiencing depression, helping to mitigate the impact of pandemic-related stress and reduce adverse postpartum outcomes [46].

Seeing the positive side of the pandemic seems to be related to the informant’s ability for resilience in terms of acceptance, adaptability, coping skills, support, and a solution-focused approach in challenging situations. The pandemic provided opportunities to rest and also spend more time with their partner and family. Individuals’ ability for resilience varies depending on personal characteristics and the contextual factors [43]. Several informants in our study expressed an ability to cope and exhibited resilience prior to the situation, often maintaining a solution-focused approach. Resilience during pregnancy has a protective role and can significantly influence both maternal and offspring outcomes [47]. Moreover, resilience during pregnancy, alongside a supportive social environment, may serve as important protective factors in crisis interventions [43].

Researchers have identified various coping phenotypes, where passive coping is associated with higher levels of depression and anxiety [23]. Factors such as job insecurity, family concerns, and media consumption are predictors of mental health outcomes [24]. However, resilience and adaptive coping strategies emerged as protective factors. These strategies included virtual communication, self-care behaviors, partner support, and maintaining routines [16,24]. Understanding these experiences and coping mechanisms is crucial for healthcare providers to support pregnant and postpartum women during times of crisis [22].

In a recent review of the impact of the COVID-19 pandemic on stress resilience and mental health, there were large interindividual differences in how the pandemic affected people’s experiences [48]. It is essential to comprehensively and interactively assess the capacity for resilience to provide person-centered care and interventions for vulnerable groups in case of future pandemics. The constantly changing restrictions during the pandemic demonstrate the need for evidence-based guidelines that are regularly updated, as well as education for professionals about how to adapt healthcare during the whole perinatal period in case of future pandemics [48]. In order to minimize the mental health consequences of COVID-19 for pregnant women, interventions should focus on bolstering coping skills, considering that coping is one pathway that impacts perinatal mental health. Structured questions to assess the capacity for resilience and coping with difficult situations would be suitable to offer individual solutions or support if needed [49].

### Strengths and limitations

The findings of the present study should be interpreted in the light of its strengths and limitations. The criteria for assessing the quality and trustworthiness of the conducted study, as described by Guba and Lincoln [40], including credibility, dependability, confirmability, and transferability, were considered. The process is described in detail to provide transparency and accurately document the method, analysis, and results to enable the reader to assess trustworthiness and transferability. Also, quotations are included to enhance trustworthiness of the analyzed data. Credibility was achieved by the purposeful sampling of informants, continuous analysis of the data, description of the entire process, and supporting quotes. To avoid lone researcher bias, three authors individually read the transcripts and conducted the initial analysis. To increase dependability, the analytical process was rigorous and systematic; all data were thoroughly analyzed. This was done to maintain awareness of personal assessments throughout the analysis process. During the development of the categories, they were repeatedly discussed by Y-LL, HN, and CÅ to ensure clear distinctions between the categories and subcategories. The results were subsequently reviewed by researchers with extensive experience in qualitative research (MG and AHE). The criteria for assessing trustworthiness were carefully considered throughout the process.

The study also has certain limitations. The interviews were conducted during the COVID-19 pandemic, and the period during which they took place was relatively lengthy, with the pandemic evolving in various ways throughout this time. Several of the pregnant women were isolated at home and uncertain about the potential effects of the coronavirus on their pregnancy and child. During the period of data collection, new developments related to the progression of the pandemic emerged. This led to the inclusion of a relatively large number of women in the study, allowing us to capture variations over time and from different perspectives. The inclusion of 30 women also provides greater opportunities to explore the diversity of experiences related to the studied phenomenon. While the sample reflects the broader cohort, caution should be exercised when transferring the findings to populations with different demographic or contextual characteristics. We have considered the potential selection bias arising from the exclusion criteria. A limitation of our study is that language barriers were used as an exclusion criterion for the ongoing study from which we recruited interview participants. This may have affected the diversity of perspectives included and could limit transferability [33] of our findings to populations who speak other languages.

### Clinical implications

The partner plays a crucial role in the woman’s well-being, and research suggest that future pandemic strategies should view the family as a unit [15]. Perinatal healthcare in Sweden typically maintains this perspective; however, during the pandemic, it was deprioritized. For the future, there is a need for assessment and interventions to best support women during the highly vulnerable perinatal period [3,16]. The need for more personalized communication is important for pregnant women to cope with uncertainty during a pandemic [14]. Organizations need to develop person-centered care strategies during pandemics and implement solutions to optimize the psychosocial well-being of pregnant women while ensuring the active participation of their partners [42]. Expectant parents represent a group for whom person- and family-centered care would be highly applicable in the event of future pandemics, making the establishment of national guidelines that prioritize consistent, accessible, and inclusive perinatal healthcare essential.

### Future research

Further research is needed to explore the long‑term implications of pandemic‑related restrictions, particularly regarding partner involvement during childbirth, and how experiences such as giving birth without a partner may affect women’s perceptions of and expectations for future pregnancies and births. It would also be valuable to examine how the absence of a partner during labor and delivery shaped women’s emotional experiences, including the potential development of traumatic birth perceptions, and how such experiences influenced the couple relationship and the partner’s opportunity to bond with the newborn in the early postpartum period. Moreover, for women who have since given birth again, future research could investigate how their pandemic‑affected birth experience informed their subsequent childbirth experiences, expectations, and outcomes. Additionally, further research to examine the experiences of pregnant women with preexisting mental health conditions is important to investigate if pandemic‑related restrictions may have intensified symptoms of anxiety, depression, or other psychiatric disorders, particularly during the postpartum period. It is also important to investigate how women who did not understand or speak Swedish were affected, as language barriers may have limited access to information, reduced opportunities for communication, and increased vulnerability during pregnancy and childbirth. Exploring these groups’ experiences would deepen understanding of the broader and longer‑term consequences of perinatal restrictions and help inform maternity care practices that promote emotional well‑being, equity, and family involvement in future public health crises.

## Conclusion

The COVID-19 pandemic profoundly disrupted the perinatal experience, posing emotional, relational, and practical challenges for pregnant women and their partners. Key issues included disrupted care, exclusion of partners, inconsistent information, and increased maternal mental health concerns. Despite these difficulties, expectant parents demonstrated resilience, with solution-focused coping strategies aiding adaptation. The study underscores the need for patient-centered, inclusive, and accessible perinatal care, particularly during crises. Its findings align with existing research and offer valuable insights for shaping future healthcare policies and practices. This study highlights the central role of partner support and clear, consistent communication in promoting women’s sense of safety during pregnancy. Restrictions on partner involvement, even when medically justified, may have unintended psychological consequences and should therefore be carefully considered in maternity care planning. Furthermore, the study provides insights that are relevant for future public health crises. Preparedness strategies should include not only infection control measures but also consideration of psychosocial support.

## Supporting information

S1 File COREQ Checklist. (PDF)

S2 File Interview guide for semi structured individual interviews. (DOCX)
