# Supplementary material for: A solo journey in the shadow of a double-edged pandemic: A qualitative study of women’s experience of being pregnant during the COVID-19 pandemic
Source: PLoS One. 2026 May 15;21(5):e0349378. doi: 10.1371/journal.pone.0349378 (PMC13178878; doi:10.1371/journal.pone.0349378)
Supplement: S2 File — (DOCX) [file pone.0349378.s002.docx]

# **Interview guide**

**Women’s experience of being pregnant during the COVID-19 pandemic.**

**Introductory questions**

- How has the COVID-19 pandemic affected you? (both in general, and during pregnancy).
- How are you now, overall?
- How is your mental health? (Follow-up questions may address anxiety, distress, depressive symptoms, or stress.

**Childbirth and the maternity hospital stay**

- What are your thoughts and feelings towards giving birth in the light of the current situation?
- Have your thoughts and feelings (possibly including worry) in the recent months (since the COVID-19 pandemic began)?
- If your partner would have cold symptoms and wouldn’t be allowed to be present during childbirth – what are your thoughts about that?
- Follow up on potential worry or fear related to childbirth, based on the woman’s responses to the Fear of Birth Scale (FOBS) questionnaire.
- Thoughts about the stay at the maternity hospital if the partner is not allowed to stay with you?

**Relations and support**

- Has the relationship to your partner changed? If yes; in what way?
- What kind of social support do you currently have? (E.g. partner, family, friends).

**The contact with the maternity clinic**

- How have you experienced the contact with the midwife/maternity since the pandemic started?
- How have you experienced the situation where your partner was not allowed to attend during the visits to maternity and possible ultrasound?
- Have you been able to participate in a parenting group? If not, what are your thoughts about that?

**Information from healthcare and authorities**

- How do you perceive the information provided by authorities and the healthcare system, both in general and specifically for pregnant women or expectant parents?

**Media coverage of the pandemic**

- How have you been affected by the media’s coverage of the pandemic?
